# Knowledge management: a practical guide for librarians

**DOI:** 10.29173/jchla29732

**Published:** 2023-12-01

**Authors:** Pamela S. Morgan

**Affiliations:** Division Head, Information Resources and Archives Health Sciences Library, Memorial University of Newfoundland, St. John’s, NL, Canada

Bartlett JA. **Knowledge management: a practical guide for librarians**. Lanham, MD: Rowman & Littlefield; 2021. Paperback: 139 p. ISBN: 978-1-5381-4457-2. Price: USD$ 69.00. Available from: https://rowman.com/ISBN/9781538144572/Knowledge-Management-A-Practical-Guide-for-Librarians

Knowledge is one of the most valuable assets any organization can have, if only the organization can manage it to make it useful. For librarians who are used to information management, knowledge management seems like something that should come naturally, a progression from managing information to managing knowledge. But knowledge can be hard to get a handle on; it includes the sum of experiences as well as documents. *Knowledge management: a practical guide for librarians* describes how a librarian can develop a knowledge management program that will allow library staff to obtain needed knowledge efficiently and effectively without duplicating effort and that will help transfer knowledge from one person to another. It would be useful for any librarian working on a knowledge management program or wanting to get an overview of what is involved in knowledge management from a library perspective.

The author is an interim associate dean at the University of Kentucky Libraries with over 20 years experience in both public and academic libraries and has written the New and noteworthy column for *Library leadership & management* for over 10 years. This is her second book, the first being an edited title on organizational knowledge in which she co-authored two chapters.

The book is an easy read of both the conceptual and practical, describing underlying theories and how a plan comes together via these theories. There are books that give more detailed explanations and examples, and others that focus more on theories. This book provides a good balance. It gives an overview of some of the models while putting them in the context of practical applications for a library. The structure of the book is centred around the framework of the knowledge management lifecycle and the challenges associated with implementing knowledge management from the point of view of people, process, and technology.

The book is laid out in three sections. The first is an introduction that goes through definitions, types of knowledge, the relationship of knowledge management to information management, and the knowledge management lifecycle. It introduces the concept that knowledge management is primarily about people. The second section goes through each of the five stages of the knowledge management lifecycle (capture, organize, store, share, and update) in greater depth, while the brief third section talks about the future of knowledge management. Each chapter provides a key to the topics to be introduced, a quote that sums up the intent of the chapter, a summary of the key points, and a reference list. The chapter references are all duplicated at the end of the book into one bibliography, which seems unnecessary. Figures are helpful in illustrating the models being discussed but are, for the most part, duplicated to highlight the stage of the knowledge management lifecycle under discussion. Tables are generally welcome and well done. There are many highlighted text blocks (sections of text set aside outside the normal flow of the text). While containing useful information, they were inconsistent, sometimes acting as footnotes, sometimes highlighting definitions or examples, sometimes containing actual exercises.

The targeted exercises are not clearly distinguishable at a glance because they have a variety of names and different formatting. They are not listed in the Table of Contents, the index, or in a separate list of exercises. It would have been helpful to be able to easily find them without having to skim through the entire book so that one could refer back to them. There is only one vignette with a link for more information, described as a case study. The book could have done with additional cases or examples. There are a couple of minor typos and grammatical errors.

One of the takeaways I had from reading this book was the relationship between Data, Information, Knowledge, and Wisdom (the DIKW pyramid) as well as the explanations of the types of knowledge. Most of us are familiar with the type of knowledge that is documentation, but the idea of capturing the knowledge that comes from experience brings a new dimension.

I was also struck by the chapter on the culture of the organization. This book stresses that knowledge management is not an over and done project. It is an ongoing process that involves a lot of people management. Knowledge is built around people’s experiences and getting them to share their hard-won knowledge that gives them an advantage over their colleagues can be a challenge.

The chapter on technology is insightful as I see a lot of the technology mentioned being used in my library system, but without an overarching plan. Knowing which system to go to for what documentation, and how easy it is to find and retrieve, can be as big an issue as knowing what documentation is available.

Published in 2021, the importance of knowledge management to any organization is highlighted in the chapter on the future of knowledge management as it relates specifically to the fallout from Covid. The increased need to be able to access and transfer knowledge as remote work comes to the forefront begs the question as to whether our documentation and knowledge is accessible from wherever we need it and whenever we need it.

This book is helpful in emphasizing how knowledge management is so much more than an individual endeavor; it is an organizational process requiring buy-in at all levels. Part of the *Practical guide* series from Rowman & Littlefield (#73), the stated scope of the book is “to offer basic background information and context … to involve readers with useful targeted exercises and examples to develop and implement a knowledge management program”. The back cover adds that the book offers “just the right balance between theory and practice *”. Knowledge management* does balance theory and practice, and it does provide basic background information, but the exercises and examples are lacking. They are not always clearly identified, and only go so far towards developing a knowledge management program. This book is a good starting point for an introduction to the topic and some of the models and for gaining familiarity with the terminology, but it cannot be the sole reference. However, it is a good launching pad to get you thinking and to identify where you need to do further exploration to get you on the road to your own program.

